# Change and stability in British drinking practices and culture between 2009 and 2019: A longitudinal latent class analysis of drinking occasions

**DOI:** 10.1016/j.ssmph.2023.101548

**Published:** 2023-11-04

**Authors:** John Holmes, Alessandro Sasso, Mónica Hernández Alava, Abigail K. Stevely, Alan Warde, Colin Angus, Petra S. Meier

**Affiliations:** a School of Health and Related Research, University of Sheffield, Sheffield, UK; b European Commission, Joint Research Center (JRC), Ispra, Italy; c School of Social Sciences, University of Manchester, Manchester, UK; d MRC/CSO Social and Public Health Sciences Unit, University of Glasgow, Glasgow, UK

**Keywords:** Drinking occasions, Drinking culture, Event-level, Trends, Drinking practices, Social practice theory

## Abstract

**Rationale:**

Theories of practice can support understanding of health-related behaviours, but few studies use quantitative methods to understand time-trends in practices. This paper describes changes in the prevalence and performance of alcohol drinking practices in Great Britain between 2009 and 2019.

**Methods:**

Latent class analyses of annual cross-sectional data collected between 2009 and 2019. The dataset come from a one-week retrospective diary survey of adults resident in Great Britain. It contains 604,578 drinking occasions reported by 213,470 adults (18+) who consumed alcohol in the diary-week. The measures describe occasion characteristics including companions, location, motivation, timings, accompanying activities and alcohol consumed. We estimate separate latent class models for each year and for off-trade only (e.g. home), on-trade only (e.g. bar) and mixed-trade occasions.

**Results:**

We identified fifteen practices; four off-trade only, eight on-trade only and three mixed-trade. The prevalence of practices was largely stable over time except for shifts away from drinking with a partner and towards drinking alone in the off-trade, and shifts away from *Big nights out* and towards other forms of heavy drinking in the on-trade. We identified five key trends in the performance of practices: (i) spirits increasingly replaced wine as the main beverage consumed in occasions; (ii) home-drinking moved away from routinised wine-drinking with meals on weekdays and towards spirits-drinking on weekends; (iii) the *Male friends at the pub* practice changed less than other pub-drinking practices; (iv) *Big nights out* started later, often in nightclubs, and involved less pub-drinking or heavy drinking and (v) the meal-based and *Going out with partner* practice formats showed few changes over time.

**Conclusion:**

Key recent trends in British drinking practices include a decline in routinised wine-drinking at home, a transformation of big nights out and a mixture of stability and change in pub- and meal-based practices.

## Highlights

• Few quantitative studies describe how drinking cultures and practices change over time.
• Drinking diary data show how British drinking culture changed between 2009 and 2019.
• People moved away from routine wine-drinking at home on weekdays.
• Big nights out started later and were less likely to involve pub-drinking.
• Traditional male pub-drinking changed less than other forms of pub-drinking.

## 1 Introduction

There is a long research tradition of studying national drinking cultures to understand and explain patterns of alcohol consumption, related health and social problems, and the politics of alcohol (Savic, Room, Mugavin, Pennay, & Livingston, 2016). Quantitative research in this tradition typically treats alcohol use as a simple behaviour and assumes each national culture can be represented by its position on a small number of dimensions (Meier, Warde, & Holmes, 2017). These dimensions include the volume and frequency of alcohol consumption in the population, the predominant type of alcohol consumed (e.g. beer, wine), and the extent to which people drink in off-trade (e.g. home) or on-trade (e.g. bar) settings. This dimensional approach is useful for identifying archetypal drinking cultures, such as the ‘wet’ Mediterranean culture and ‘dry’ Scandinavian culture (Savic, Room, Mugavin, Pennay, & Livingston, 2016), but it has five important limitations. First, it assigns a single drinking style to each culture and disregards differences within cultures. Second, it offers little definitional clarity for cultures that lie between archetypes. Third, the behaviours of the highest consuming groups (e.g. men) play a disproportionate role in defining the culture as their drinking behaviours dominate average values in national statistics (Ally et al., 2016). Fourth, the dimensional approach limits our ability to understand change in national drinking culture as transitions from one major archetype to another are rare (Room, 1992), whereas smaller changes may be more common. Finally, the simplicity of the dimensions offers little opportunity to understand or test the mechanisms by which drinking cultures change.

We have previously argued that theories of practice provide an alternative conceptual framework that allows more nuanced analyses of drinking culture (Meier, Warde, & Holmes, 2017). We have used this framework to develop a typology of drinking occasions that offers a detailed quantitative description of British drinking culture (Ally et al., 2016), as well as exploring the characteristics of heavy drinking occasions (Stevely, Holmes, & Meier, 2021). In the present paper, we demonstrate how a practice approach can offer further detailed insights when used to examine changes over time in drinking culture.

### 1.1 Theories of practice

Our practice-based approach draws on Shove et al.‘s account of social practice theory as its accessibility and clear ontology support its use in quantitative research (Shove, Pantzar, & Watson, 2012). We argue that quantitative analyses can view national drinking cultures as comprising a set of drinking practices that we define as recognisable and distinct ‘types’ of occasion in which people consume alcohol. For our purposes, the term ‘occasion’ simply denotes a period of time rather than a noteworthy event. Drinking practices in turn comprise unique combinations of practice elements. Shove et al. allocate elements to three categories: materials (e.g. alcoholic drinks, glassware, buildings), competencies (e.g. brand awareness, etiquette, alcohol tolerance) and meanings (e.g. sophistication, relaxation, transgression). Some relevant elements of drinking practices do not fit neatly within these categories (MacLean et al., 2019), such as the temporalities of drinking (e.g. duration, time, day), which play an important role in shaping and differentiating how people drink (Southerton, 2013). The categories are therefore best viewed as a framework for analysing the specific elements or combinations of elements that confer symbolic significance on groups of similar occasions and thus denote different occasion types. Analysts may identify greater or fewer numbers of occasion types. Some of these may be widely recognised and engender shared norms when in a relevant situation or context (e.g. big nights out, romantic meals), but others may be more amorphous, reflecting looser combinations of elements or weaker normative influences (e.g. relaxed home drinking). We therefore refer mainly to occasion types as ‘practice formats’ rather than practices per se, recognising the differing degrees of normative influence (Warde, 2016).

Practice theories often struggle to explain change as they necessarily focus on routines and habits that tend towards stability over time (Warde, 2016). Shove et al. (2012) argue that practices achieve stability by individuals acting as carriers of ‘practice entities’ (i.e. the socially recognisable form of a practice) that are sustained through repeated ‘performances’ (i.e. individual instances of a practice). Other factors also play a role in supporting stability, such as synergistic relationships with other established practices and formal or informal articulations of appropriate standards of practice (e.g. manuals, critical reviews, peer pressure) (Warde, 2016). Conversely, change arises through variations in performance that disrupt practice entities. This includes individuals undertaking performances that adapt the combinations of elements that form a practice, for example adding or removing elements or reconfiguring the relationships between them (Shove, Pantzar, & Watson, 2012). This may arise from deliberate attempts to innovate (Warde, 2016). It may also arise from technological developments, policy interventions, changes in closely related practices, or wider shifts in the practices of everyday life as each of these may alter the availability of elements and the opportunities to combine them (Hargreaves, 2011; Maller, 2015; Shove, 2010). Changes in practice performance can then spread across a population and cause practice entities to evolve in form. In some cases, new entities may emerge as existing ones split, combine or transform. Processes of change do not only affect the practices that exist in a society but also how often they are performed as practices must compete with each other for finite elements (e.g. space, time, desirable meanings). As such, practices may increase or decrease in prominence, and potentially disappear, as they lose the carriers that sustain them or carriers perform the practice less often.

A growing body of public health research draws on theories of practice to gain new insights into health risk factors (e.g. Blue et al., 2014; Cohn & Lynch, 2017; Maller, 2015). For example, several studies seek to identify and classify the elements involved in particular health-related practices, such as alcohol use, cycling, gambling, snacking and vaping (Hennell, Piacentini, & Limmer, 2021; Keane, Weier, Fraser, & Gartner, 2017; Nyemcsok, Pitt, Kremer, & Thomas, 2022; Spotswood, Chatterton, Tapp, & Williams, 2015; Twine, 2015; Wright, Miller, Kuntsche, & Kuntsche, 2022). Hennell et al. take this approach further for alcohol by unpacking the ‘bundles’ of smaller practices that comprise the larger practice of a ‘Proper Night Out’ (Hennell, Piacentini, & Limmer, 2020). These include planning, getting ready, pre-drinking, going out, getting home and storytelling. Other work uses theories of practice to evaluate or explain the impact of public health interventions or health-related smartphone apps (Cohn & Lynch, 2017; Spotswood, Shankar, & Piwek, 2020), to explore drivers of health inequalities (Spotswood & Gurrieri, 2022) or to gain insights into the health-related experiences of marginalised groups (MacLean et al., 2019; Spotswood et al., 2021). However, few studies use quantitative methods or analyse change over time in the practices associated with a major health risk factor (see Mäkelä et al., 2022 for an example). Doing so offers new possibilities to classify the different ways people engage with health risk factors and changes in the extent and nature of this engagement over time.

### 1.2 Alcohol drinking practices

Alcohol consumption is a useful case study for studying practice changes as it has displayed marked trends in recent years. Litres of pure alcohol consumed per capita reached a historic peak in 2004 but then fell by 16% to 2019 (Public Health Scotland, 2022). Several significant, and sometimes countervailing, changes underpinned this fall. For example, reduced beer drinking in pubs drove much of the decline in alcohol consumption, leading to the closure of many community pubs in deprived areas (Angus et al., 2017). The later emergence of a largescale craft beer and cider market led to a partial recovery but was associated with more affluent areas (Thurnell-read, 2018). In contrast, levels of home-drinking remained relatively stable and public health concerns rose in the UK and around routinised wine-drinking, popularly referred to internationally as wine o'clock. The sharp decline in youth drinking was another key driver of change (Oldham & Holmes, 2018), but exhibited some complexity as pre-drinking at home before a night out emerged as a popular way to save money, socialise in a relaxed setting and prepare for the challenges of the night-time economy (Hennell et al., 2020). A practice lens permits more nuanced quantitative description and, potentially, explanation of these trends, their uniformity (e.g. whether all or only some home- and pub-drinking changed) and the drinking culture that emerged from them.

In our previous cross-sectional analyses of British drinking culture (Ally et al., 2016), we used latent class models of a large and detailed diary survey dataset to describe a typology of drinking occasions (e.g. *Big night out*, *Family meal*). We argue that drinking occasions recorded in the diaries are observations of practice performances while the occasion types (i.e the latent classes) describe the prevalence and characteristics of practice formats. The present analysis builds on this approach by using extensions of the same dataset to estimate comparable latent class models for each year of our study period. This allows us to describe changes in the prevalence and characteristics of practice formats. Theoretically, it also allows us to detect new formats, although we did not anticipate doing so over our relatively short eleven-year study period.

This paper therefore aims to describe changes in the prevalence and performance of the practice formats that comprise British drinking culture between 2009 and 2019.

## 2 Methods

### 2.1 Data

We used data from Alcovision, a continuous, cross-sectional study that has surveyed approximately 30,000 adults (18+) resident in Great Britain each year from 2009 onwards using largely consistent methods. The market research company Kantar collects Alcovision data for commercial purposes and draws quota samples based on age, sex, socioeconomic status and geographic region from its online managed access panels. It times invitations to participate to spread fieldwork evenly throughout the year and include every day of the year. Residents of Scotland and 18-34 year-olds are oversampled to allow detailed analyses of these populations. Kantar then constructs sampling weights based on age-sex groups, social class and geographic region using UK census data. The present analysis used updated weights constructed by the authors using a bespoke raking technique (see Appendix A). The main data collection changes are a switch to sampling from additional panels from 2017 onwards and the loss of all data for July 2017. We address these limitations in the discussion section.

The main component of Alcovision is a retrospective drinking diary, in which participants report the characteristics of their drinking occasions over the previous seven days. This study used Alcovision data from 2009 to 2019, which includes 1,042,942 drinking occasions. However, we combined some closely-related occasions where individuals moved between off-trade and on-trade drinking (see Section 2.3. Analysis), so the analytical sample is 604,578 occasions reported by 213,470 individuals who consumed alcohol in the diary week.

### 2.2 Measures

Alcovision defines drinking occasions as periods when participants consumed alcohol that are separated by a significant time-period (e.g. lunchtime, late evening). It divides these occasions into two trade sectors: those in the off-trade (e.g. at home) and on-trade (e.g. at a pub or restaurant), with participants able to report up to two occasions in each trade sector per day. Alcovision's division of occasions into off-trade and on-trade does not allow for direct analysis of familiar occasions that span both trade sectors (e.g. pre-drinking before a night out). We therefore used an alternative definition based on the start-time and duration of each reported occasion. This defines occasions as a period of alcohol consumption with no more than 2 h between drinks and allowed us to combine temporally adjacent occasions within and across trade sectors. We then separated occasions into three group for all analyses: off-trade only, on-trade only and mixed-trade.

Participants record the characteristics of each reported drinking occasion via a series of categorical variables that did not change across the study period. As noted in the introduction, these do not fit neatly into Shove et al.’s (2012) domains of materials, competencies and meanings (see Ally et al., 2016 for further discussion of this). However, they do generally fit with domains described within wider practice theories (Schatzki et al., 2001). The characteristics measured or response categories within measures sometimes differ across trade sectors, so we summarise the measures below and describe them fully in Appendix B (Table B1).

**Table 1:** Summary statistics for selected drinking occasion characteristics in Great Britain, 2009–2019.

		% of occasions with characteristic										
	N	2009	2010	2011	2012	2013	2014	2015	2016	2017	2018	2019
All	*604,578*	*64,311*	*61,232*	*59,988*	*60,404*	*57,631*	*57,196*	*55,663*	*54,350*	*44,276*	*45,984*	*43,543*
***Trade sector***												
Off-trade	417,219	69.7	71.3	70.1	68.7	68.8	68.1	68.0	67.9	68.3	68.6	68.9
On-trade	121,581	19.9	19.2	19.9	20.4	19.8	20.3	20.2	20.1	20.3	20.7	20.8
Mixed	65,778	10.4	9.5	10.0	10.9	11.4	11.6	11.8	12.0	11.4	10.7	10.3
***Sex composition***												
Mixed sex group	220,065	36.7	36.3	37.5	37.2	36.4	36.7	36.2	35.6	34.6	35.5	37.0
Mixed sex pair	212,203	37.3	37.2	36.3	36.0	36.0	35.5	34.7	33.7	32.8	32.8	31.3
Male alone	65,006	9.1	9.2	9.2	10.0	10.8	10.8	11.3	11.9	13.5	12.8	11.6
***Companions***												
Partner	275,586	46.7	47.0	47.5	46.3	45.4	45.6	45.4	44.3	44.0	44.0	43.5
Friends	165,114	27.5	26.4	27.8	27.7	27.2	27.4	27.6	28.2	27.3	26.1	26.9
Family	132,833	22.7	22.8	22.5	22.5	23.2	21.8	22.0	21.6	20.2	21.0	20.1
***Venue***												
Own home (off-trade)	400,077	65.7	67.5	65.8	65.7	66.5	66.1	66.0	66.2	66.4	66.5	65.7
Other's home (off-trade)	70,195	12.3	11.7	12.4	12.4	11.9	11.7	11.8	11.6	11.1	9.8	10.0
Traditional pub (on-trade)	65,649	10.8	10.8	10.4	11.3	10.6	11.1	10.5	10.8	10.9	11.1	11.3
Modern bar (on-trade)	31,904	4.9	4.9	4.8	5.1	5.3	5.4	5.6	5.6	5.9	5.5	5.7
Multiple venues (on-trade)	25,404	4.2	3.8	4.2	4.3	4.0	4.1	4.2	4.1	4.2	4.4	4.9
***Purpose of occasion***												
Quiet drink (off-trade)	146,289	22.0	23.2	23.3	24.2	24.3	23.9	25.2	26.2	25.9	24.2	24.8
Regular drink (off-trade)	146,045	24.7	24.8	24.4	23.5	23.4	23.9	23.6	23.8	23.7	25.7	24.6
Other (on-trade)	84,619	13.1	12.6	13.0	14.1	14.1	14.6	14.6	14.7	14.6	14.4	14.8
Sociable (on-trade)	42,182	7.4	6.9	7.1	7.0	7.1	7.0	7.1	7.0	6.9	6.6	6.4
***Food eaten***												
None	222,302	33.8	36.1	35.2	36.4	36.1	36.6	36.7	37.3	38.8	40.2	39.7
Snack	95,514	15.7	14.9	16.3	16.9	15.9	15.8	16.0	16.1	15.0	15.6	15.3
Meal	286,762	50.5	49.0	48.5	46.8	48.1	47.6	47.2	46.6	46.2	44.3	45.0
***Duration***												
Less than 1 h	175,623	32.0	32.4	30.7	29.3	30.0	28.7	28.2	27.0	27.1	25.5	25.9
1–4 h	340,749	54.6	54.6	55.1	55.8	55.6	56.0	56.0	57.1	57.5	60.1	59.9
4–7 h	72,844	11.2	10.9	11.8	12.3	11.8	12.2	12.7	13.0	12.6	12.4	12.1
***Start-time***												
Evening	389,192	66.5	66.1	66.2	65.0	64.8	63.9	64.0	64.3	64.1	61.1	60.0
Afternoon	106,111	15.8	15.9	16.1	17.1	17.2	17.5	18.0	18.0	18.3	20.3	20.8
Lunchtime	50,926	8.7	8.9	8.5	8.7	8.6	8.3	8.1	8.3	7.9	8.1	8.3
***Day of week***												
Mon – Fri (17:00)	242,038	42.0	41.7	41.0	40.9	40.3	39.5	39.1	39.0	38.6	38.3	38.3
Fri (17:00) – Sat	261,911	40.9	41.2	42.1	42.3	42.8	43.7	44.4	44.4	45.1	45.8	46.2
Sunday	100,629	17.1	17.1	16.9	16.8	16.8	16.8	16.5	16.6	16.4	15.9	15.5
***Units of alcohol consumed***												
0.0–2.0	96,497	16.0	16.4	15.8	15.0	15.6	15.5	15.8	15.0	16.4	18.0	16.8
2.0–3.5	122,382	20.6	20.8	20.4	19.8	20.8	20.8	20.8	21.2	19.5	18.6	18.4
3.5–5.0	86,107	14.8	14.4	14.4	14.4	14.6	14.2	13.9	14.1	13.8	13.8	13.8
5.0–12.0	194,805	32.8	33.1	32.9	33.3	32.2	32.3	31.7	31.6	32.3	30.7	30.6
12.0–20.0	60,709	9.5	9.1	9.6	10.2	9.9	10.0	10.4	10.5	9.9	10.6	11.3
>20.0	44,078	6.3	6.2	6.9	7.3	6.9	7.2	7.3	7.7	8.1	8.3	9.1
***Predominant beverage***												
Beer	211,297	34.6	34.4	34.5	35.5	34.2	34.7	34.8	35.0	35.7	36.6	35.0
Wine	206,140	38.8	37.7	37.4	34.6	34.3	33.7	33.3	32.5	30.8	28.9	28.9
Spirits	120,314	18.4	19.2	18.9	19.5	19.8	19.4	19.6	19.8	20.6	21.8	23.6

^1^Selection of variables based on authors' assessment of their importance. Selection of categories within variables based on the highest prevalence categories within each variable and trade sector. See Appendix B, Table B1 for full data. Categories labelled off-trade or on-trade relate to those trade sectors only.

Two measures capture the people present: *sex composition* (e.g. male alone, female pair) and *companions* (e.g. family, friends). Four measures capture the locations: the *venue* (e.g. own home, modern bar), *on-trade venue locations* (e.g. village or rural, city centre), the *reasons for choosing on-trade venues* (e.g. friendly atmosphere, cheap) and a dichotomous variable for visiting *multiple on-trade venues*. Two measures capture the nature of the occasion: the *purpose* (e.g. quiet night in, going clubbing) and the *motivation* for the occasion (e.g. to wind down, to have a laugh). Two measures capture activities accompanying drinking: *general activities* (e.g. watching TV, doing a pub quiz) and *food eaten* (no food, a snack, a meal). Four measures capture the occasion timings: the *duration* (e.g. <1 h, 4–7 h), the *start-time* (e.g. lunchtime, night-time), the *day of the week* (i.e. Monday to 17:00 on Friday, 17:00 on Friday to 23:59 on Saturday, Sunday) and the *sequencing* of off-trade and on-trade drinking in mixed-trade occasions (e.g. pre-drinking [off then on], post-loading [on then off]). Two measures capture alcohol consumption: *volume consumed* based on units (1 UK unit = 8 g ethanol; e.g. 0.0–2.0 units, 5.0–12.0 units) and the *predominant beverage type* based on the largest number of servings reported (e.g. beer, wine).

### 2.3 Analysis

Latent class analysis is a statistical method used to identify distinct subgroups of cases in a dataset based on them having similar patterns of observed characteristics. It assumes that a latent (i.e. unobserved) categorical variable explains the correlation between a set of observed variables. The estimated model describes the probability of a case belonging to a specific subgroup (i.e. latent class) and, conditional on belonging to a subgroup, the probability of it having each observed characteristic. In this analysis, drinking occasions (i.e. practice performances) are the cases, their characteristics are the observed variables and occasion types (i.e. practice formats) are the subgroups or latent classes.

We estimated separate latent class models for off-trade only, on-trade only and mixed-trade sectors in each year, using the occasion characteristics above. Estimating separate models for each trade sector was necessary given the occasion characteristic variables differ across trade sectors. Latent class models estimate the joint distribution of all of the observed variables and the latent class variable under the assumption of conditional independence. The association between the observed and latent variables are parameterised using probits, ordered probits and multinomial logits for binary, ordered and unordered categorical variables respectively, whereas the probability of belonging to a specific class is parameterised using a multinomial logit. The analyst iteratively determines the number of classes the model should identify by assessing the impact of additional classes on model fit and interpretability. All models used weighted data and accounted for the clustering of occasions within individuals using clustered standard errors. The latent class models were estimated via maximum likelihood using Mplus v8.3.0.1.

We first examined the model fit statistics and interpretability for a series of preliminary LCA models for each year to assess whether the appropriate number of latent classes differed across years, whether the identified classes were broadly consistent, whether some classes emerged or disappeared over time, or whether the model results were fundamentally unstable across years. Specifically, we used the Bayesian Information Criterion (BIC), Sample-Size Adjusted BIC, Akaike's Information Criterion, and model entropy as standard goodness of fit measures, and also used the Vuong-Lo-Mendell-Rubin likelihood ratio test (LRT), the Lo-Mendell-Rubin adjusted LRT and the parametric bootstrapped LRT to test whether each additional class improved model fit. These tests require careful interpretation as they are sensitive to the sample size and number of model parameters, both of which are large in Alcovision (Tein et al., 2013). We assessed interpretability using the authors' topic expertise and, at later stages, consulted our project's advisory group. As the models identified broadly similar classes in each year, we restricted future models to estimate the same number of classes in each year and focused our analyses on changes over time in the proportion of occasions within each class and the characteristics of those occasions.

We tested several approaches to analysing change in drinking practices over time. These included estimating a single latent transition model for each trade sector covering the whole study period, estimating several latent transition models covering shorter time periods, and estimating separate latent class models for each year and then using pairwise comparisons to test for significant differences in model parameters between years. However, these approaches encountered problems with insufficient computational power, unfeasible model run-times, and the large sample size meaning even very small differences between parameters were statistically significant despite having little practical significance.

The results reported below are therefore based on a simpler approach in which we estimated separate latent class models for each year between 2009 and 2019 and visually inspected the results to identify changes of practical significance. We defined practical significance as any sustained change of five percentage points in either the proportion of occasions within a latent class or the probability of a latent class having a particular characteristic. We defined sustained change as a change observed for three years and maintained to 2019. Applying this definition of practical significance required some subjective judgements for changes at the end of the study period, where parameters were unstable (see Section 3.2), or when smaller changes merited attention as part of a wider phenomenon that was evidenced more clearly in other larger changes. The numerical changes reported below are between 2009 and 2019 except for mixed trade occasions where we use 2009 to the average of 2016 and 2017 due to instability in the model results for later years.

## 3 Results

### 3.1 Descriptive statistics

Table 1 provides summary statistics by year for selected variables (see Appendix B, Table B1 for all variables used in the latent class models). The proportion of occasions within each trade sector was largely stable between 2009 and 2019. Overall, 69.0% (Range: 67.9%–71.3%) of occasions were off-trade only, 20.1% (Range: 19.2%–20.8%) were on-trade only and 10.9% (Range: 9.5%–12.0%) were mixed-trade.

The proportion of drinking occasions with each characteristic was also mostly stable over time, with changes of less than three percentage points (pp) between 2009 and 2019 in drinking venues, reason, purpose, motivations and most accompanying activities. The main changes related to drinking companions, food, timings and alcohol consumption. The proportion of occasions involving a mixed sex pair decreased by 5.9pp and the proportion involving a partner decreased by 3.2pp. The proportion of occasions involving no food consumption increased by 5.8pp, largely due to a 5.5pp decrease in the proportion involving a full meal. The proportion of occasions lasting less than 1 h decreased by 6.1pp, the proportion starting in the afternoon increased by 5.0pp, the proportion on a weekday decreased 3.7pp while the proportion on Friday night or Saturday increased by 5.3pp. Broadly, these temporal shifts suggest a move away from short, weekday drinking occasions, which typically start in the evening, and towards longer weekend occasions, which are more likely to start in the afternoon. The largest change was a 9.9pp drop in the proportion of occasions where wine was the dominant beverage. This contrasts with increases in the proportion of occasions where spirits and cider were the dominant beverage of 5.2pp and 3.8pp respectively, with only marginal changes for beer. Finally the proportion of occasions where individuals consumed 12 or more units increased by 4.6pp.

### 3.2 Latent class estimation results

We estimated models with between two and eight latent classes for each trade sector (see Appendix A for model fit statistics and reporting of model selection). The final models had four classes for off-trade only occasions, eight classes for on-trade only occasions and three classes for mixed-trade occasions. The off-trade only model shows little volatility over time (i.e. there are no large fluctuations that appear due to analytical processes or noise in the underlying data rather than changes in drinking practices). However, the on-trade only and mixed-trade models show greater volatility, likely due to the smaller number of occasions in these trade sectors. In particular, the mixed-trade model includes marked disconnects between years towards the end of the study period. This limits the interpretability of some aspects of these models, and we address this further in the limitations section of the discussion. When reporting time-trends below, we either do not seek to interpret changes across volatile periods, omit the outlier years from consideration where the model reverts to interpretability in later years, or suggest only cautious interpretations. We note this where appropriate in the text.

The model results are necessarily extensive given the number of occasion characteristics included and classes identified. We therefore present three sets of information. First, a narrative summary of the key findings with accompanying figures in Section 3.3 below. Second, a summary of the practically significant changes for each class in Table 2, Table 3, Table 4, including the numerical values for the figures. Third, the full model results in Appendix B, Tables B.2 to B.4. We recommend readers focus first on the narrative summary and figures and then use the summary and full tables to gain additional insights where required. For readers interested in more detailed description of the fifteen latent classes, Holmes et al. (Forthcoming) provides a full description for the 2019 model and Appendix A here provides a summary.

**Table 2:** Percentage point changes in the prevalence and characteristics of off-trade only occasion types, 2009–2019^a^.

Occasion type	% of occasions: All (off-trade)^b^			Companions	Venue	Reason, purpose and motivation	Accompanying activities (including food)	Timing	Consumption
	2009	2019	Diff.						
Quiet drink at home alone	15.7 (22.5)	19.6 (28.4)	+3.9 (+5.9)	No change	No change	Wind down (−5.3)	Chores or online shopping (−5.2) No food (+8.9) Meal (−8.6)	<1 h (−6.4) Afternoon (+5.5) Evening (−5.0) Weekday (−5.4) Fri/Sat (+5.9)	Wine (−10.1)
Family time at home	10.3 (14.8)	9.2 (13.4)	−1.1 (−1.4	Family (−12.1) Partner (+12.5)	No change	Quiet drink (+7.5)	No food (+8.9) Meal (−8.5)	<1 h (−10.1) 1–4 h (+8.0) Weekday (−5.6) Fri/Sat (+10.3)	Wine (−13.1)
Evening at home with partner	28.9 (41.4)	23.5 (34.1)	−5.4 (−7.4)	No change	No change	No change	No food (+7.1) Meal (−7.4)	<1 h (−8.1) 1–4 h (+6.5) Afternoon (+5.3) Evening (−5.2) Weekday (−6.1) Fri/Sat (+7.0)	Wine (−7.2)
Off-trade get together	14.8 (21.3)	16.6 (24.1)	+1.8 (+2.9)	No change	Own home (+9.2) Other home (−14.2)	No change	Chores or online shopping (+10.7) Online leisure (+8.8)	<1 h (−8.1) Evening (−5.6)	Wine (−16.7) Spirits (+7.2) Cider (+6.9)

a Numbers in Diff. (difference) column and occasion characteristics columns are percentage point changes. Changes greater than five percentage points shown. Some names of characteristics have been abbreviated for space reasons. Changes in ‘other’ categories within characteristics are not shown as they are uninformative.

b Percentage of all occasions outside brackets and percentage of off-trade only occasions inside brackets.

**Table 3:** Percentage point changes in the prevalence and characteristics of on-trade only occasion types, 2009–2019.^a^

Occasion type	% of occasions: All (on-trade)			Companions	Venue	Reason, purpose and motivation	Accompanying activities (including food)	Timing	Consumption
	2009	2019	Diff.						
Meeting friends at the pub	3.2 (16.3)	3.6 (17.2)	+0.3 (+0.9)	Friends (−12.5) Partner (+6.5)	Social or WMC (−7.8) City centre (+9.7) Small town (−5.1) Village/rural (−5.3) Regular/local (−9.6) Know people (−8.4)	Sociable (−11.3) Have a laugh (−7.8)	Games machine (−8.0) Other (+7.6) No food (+5.2) Snack (−7.3)	Afternoon (+6.7) Evening (−10.0) Fri/Sat (+6.3)	12–20 units (−6.8) Beer (−12.2) Spirits (+5.5)
Male friends at the pub	2.4 (11.9)	2.7 (12.9)	+0.3 (+1.0)	No change	Pub restaurant (+5.2) Social or WMC (−5.8) City centre (+6.4) Village/rural (−5.9) Convenient (+10.3) Cheap (+6.5) Feel at home (−5.2) Know people (−6.3) Other (−5.6)	No change	Watching TV (+5.2) Games machine (−7.4)	Weekday (−5.8) Fri/Sat (+10.7)	No change
Quiet drink at the pub	3.4 (17.0)	3.0 (14.2)	−0.4 (−2.8)	Mixed group (−12.2) Male alone (+11.3) Friends (−5.4) Other (−10.2)	City centre (+11.2) Village/rural (−7.5) Convenient (−7.9) Cheap (+9.0) Feel at home (−8.9)	Making time (+6.7)	Watch TV (+6.2)	<1 h (−10.5) 1–4 h (+7.2) Weekday (−7.4) Fri/Sat (+5.6)	2.0–3.5 units (−6.6)
Big night out	2.1 (10.7)	1.3 (6.3)	−0.8 (−4.4)	Mixed pair (−7.6) Male group (−6.0) Female group (+7.8) Partner (−10.9) Friends (+5.7) Other (−7.3)	Small town (−8.2) Nightclub (20.7) Traditional pub (−21.4) Modern bar (−13.6) Restaurant (−7.4) Multiple venues (−13.3) Convenient (−17.9) Quality (−11.4) Local (−8.9) Friendly (−10.0) Feel at home (−8.0) Know people (−12.0) Quiet (−6.6) Other (−16.7)	Clubbing (+13.1)	Watch TV (−9.0) Dance/music (+12.8) Pool/darts etc. (−5.4) Games machine (−13.1) No food (+17.1) Meal (−16.7)	1–4 h (+22.6) 4–7 h (−17.1) 7+ hours (−6.7) Afternoon (−10.0) Evening (−13.5) Night-time (+27.8)	0.0–2.0 units (+5.9) 3.5–5.0 units (+6.7) 12–20 units (−10.5) 20+ units (−8.9) Beer (−21.3) Spirits (+27.1)
Extended occasion (on-trade) *From 2011-2019*^b^	2.2 (7.5)	3.0 (14.6)	+0.9 (+7.1)	Partner (−7.2) Friends (−13.3) Child (+14.3)	Large town (+7.8) Edge of town (+7.9) Nightclub (+10.3) Traditional pub (+12.4) Modern bar (+12.0) Quality (+8.6)	Night out (−10.8) Live event (−8.5) Refresh (+8.4) Let go (+6.1) Have a laugh (−8.0)	Watch TV (+6.7) Pool/darts etc. (+16.2) Jukebox etc. (+13.3)	Afternoon (+5.7) Evening (−10.4) Weekday (+12.6) Fri/Sat (−14.3)	20+ units (+6.0) Beer (−9.0) Wine (−7.5) Spirits (+5.5) Cider (+7.2)
Family meal	2.0 (10.3)	2.3 (11.2)	+0.3 (+0.9)	Partner (+8.0) Family (−8.0)	City centre (+7.9) Village/rural (−10.4) Friendly (−5.1)	No change	No food (+7.2) Meal (−11.8)	Afternoon (+5.7) Evening (−7.2)	0.0–2.0 units (+6.9) Beer (−7.8) Spirits (+7.5)
Meal with friends	2.5 (12.7)	2.3 (11.1)	−0.2 (−1.7)	Female group (+6.6) Friend (+13.8)	Large town (−5.9) Pub restaurant (−7.0) Convenient (−14.1) Quality (−11.0) Lively (7.2)	Sociable (−8.3) Part of event (+5.8) Bond (+7.6) Other (−5.2)	Dance/music (+7.7) No food (+8.5) Meal (−11.7)	Lunchtime (−15.4) Afternoon (9.1) Weekday (−10.1) Fri/Sat (+9.2)	0.0–2.0 units (−5.4) 2.0–3.5 units (+5.4) Wine (−10.8) Spirits (+8.7) Cider (+7.3)
Going out with partner	2.7 (13.6)	2.6 (12.6)	−0.1 (−1.0)	No change	Village/rural (−5.7) Restaurant (−6.8) Convenient (−8.1) Cheap (+7.7)	No change	No food (+8.9) Meal (−8.8)	Afternoon (+8.9) Evening (−11.1) Weekday (−9.3) Fri/Sat (+7.5)	Beer (−5.1) Wine (−6.0) Spirits (+6.4)

^3^Shorter time period used due to instability in the model for some years.

a Numbers in Diff. (difference) column and occasion characteristics columns are percentage point changes. Changes greater than five percentage points shown. Some names of characteristics have been abbreviated for space reasons. Changes in ‘other’ categories within characteristics are not shown as they are uninformative.

b Percentage of all occasions outside brackets and percentage of on-trade only occasions inside brackets.

**Table 4:** Percentage point changes in the prevalence and characteristics of mixed-trade only occasion types, 2009–2019.^a^

Occasion type	% of occasions: All (mixed trade)^c^			Companions	Venue	Reason, purpose and motivation	Accompanying activities (including food)	Timing	Consumption
	2009	2019	Diff.						
Big night out with preloading *From 2009-average of 2016-2017*^b^	2.7 (25.9)	2.2 (18.8)	−0.5 (−7.0)	No change	Off→On (+6.2) Modern bar (−7.0) Multiple venues (−7.1) Convenient (−5.4) Cheap (+8.4) Know people (−7.2) Sport (−7.6) Lively (−7.8)	Pre-drinking (+7.5) Bond (+7.4)	Watching TV (−7.7) Games machine (−6.1) No food (+8.4) Snack (−5.2)	No change	Spirits (+6.6)
Quiet drink at home and with friends in the local *From 2009-average of 2016-2017*^b^	3.6 (35.0)	5.0 (43.2)	+1.4 (+8.2)	Mixed group (−5.0) Mixed pair (−5.2) Friends (−9.1) Child (+5.2)	Off→On (+7.8) On→Off (−6.9) City centre (+7.3) Village/rural (−6.2) Traditional pub (−8.9) Restaurant (+5.6) Regular/local (−7.7)	Sociable (−5.9) Family event (+5.6) Refresh (+6.2)	Watching TV (−9.4)	Weekday (−9.5) Fri/Sat (10.7)	No change
Extended occasion (mixed trade) *From 2009-average of 2016-2017*^b^	4.1 (39.1)	4.4 (37.9)	+0.4 (−1.2)	Mixed paid (−9.1) Male pair (+7.3) Friends (+15.9)	Off→On (−5.9) Off←→On (+8.5) City centre (+10.3) Small town (+9.7) Village/rural (−7.8) Other home (+6.5) Modern bar (+12.1) Multiple venues (+5.9) Convenient (−6.0) Sports/music (+5.6) Other (−5.8)	Sociable (+6.5) Quiet drink (+5.7) Have a laugh (+10.5) Bond (+7.1) Refresh (+6.5) Let go (+7.9)	Leisure (+13.1) Online leisure (+16.3) Chores (+9.7) Dance/music (+10.1) Pool/darts etc. (+6.4) Games machine (+6.0)	1–4 h (−18.5) 4.7 h (+9.6) 7+ hours (+9.7) Afternoon (+7.2) Evening (−7.3)	5–12 units (−10.4) 20+ units (+15.5) Wine (−13.3) Spirits (+8.7) Cider (+9.1)

a Numbers in Diff. (difference) column and occasion characteristics columns are percentage point changes. Changes greater than five percentage points shown. Some names of characteristics have been abbreviated for space reasons. Changes in ‘other’ categories within characteristics are not shown as they are uninformative.

b Shorter time period used due to instability in the model for some years.

c Percentage of all occasions outside brackets and percentage of mixed-trade only occasions inside brackets.

### 3.3 Change in drinking practices over time

The results below first describe change between 2009 and 2019 in the share of occasions accounted for by each occasion type. They then focus on four key patterns of stability and change in drinking practices: changes in the dominant beverage type, shifts away from routine wine-drinking at home, stability and change within pub-drinking, and transformation of big nights out.

**Fig. 1:**
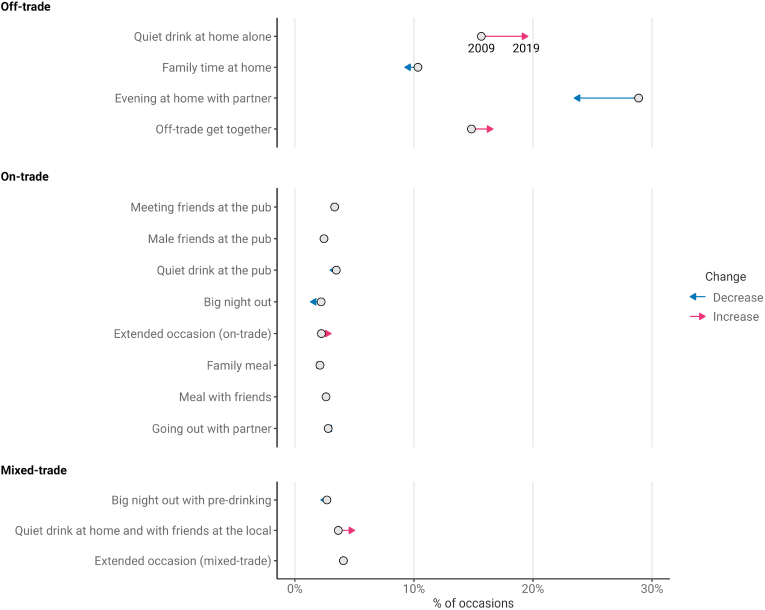
Proportion of all occasions within each type in 2009 and 2019.

Fig. 1 summarises the proportion of occasions that each type accounted for across the study period (see Appendix A for proportions by trade sector). Most occasion types accounted for a fairly stable proportion of occasions over time, including *Male friends at the pub* (+0.3pp), *Meals with friends* (−0.2pp) and *Going out with partner* (−0.1pp). However, there was a shift in the off-trade away from *Evening at home with partner* (−5.4pp) and *Family time at home* (−1.1pp) and towards *Quiet drinks at home alone* (+3.9pp). Similarly, there was a shift away from *Big nights out* (−0.8pp) and *Big nights out with pre-drinking* (−0.5pp) and towards other forms of heavy drinking occasion such as *Extended occasions (on-trade)* (+1.5pp) and *Quiet drink at home and with friends at the local* (+1.4pp).

**Fig. 2:**
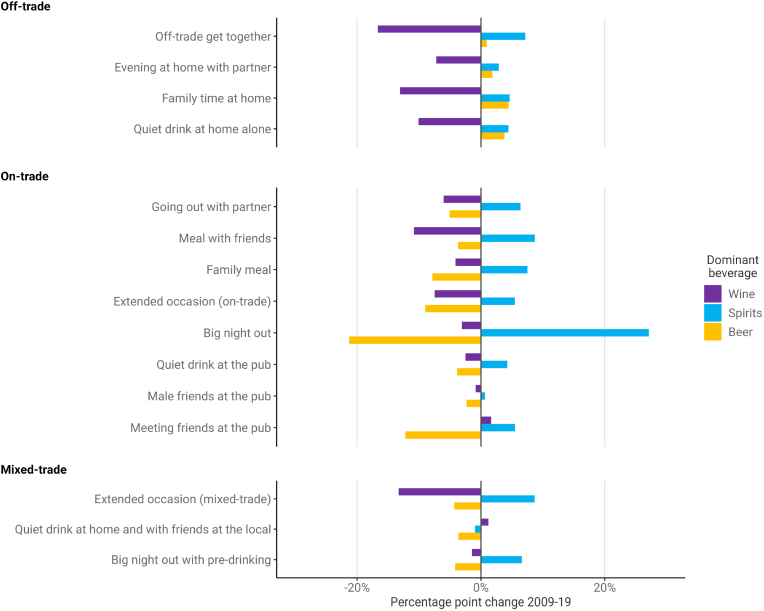
Change in the proportion of occasions where each beverage is dominant (i.e. most units consumed) by occasion type, 2009–2019.

The dominant beverage within occasions changed markedly across the study period (Fig. 2). The proportion of occasions where spirits were the dominant beverage increased in all but one occasion type and increased by more than five percentage points in nine out of fifteen types. The largest percentage point increases for spirits were for *Big nights out* (+27.1pp), *Extended occasions (on-trade)* (+13.8pp) and *Extended occasions (mixed-trade)* (+8.7pp). Conversely, the proportion of occasions where wine was the dominant beverage decreased in all but two occasion types, with large decreases seen in all off-trade only types. The proportion of occasions with beer as the dominant beverage changed less in the off-trade but did decrease substantially in some on-trade occasion types, including *Big nights out* (−21.3pp) and *Meeting friends at the pub* (−12.2pp). Some types showed only small changes in the dominant beverage, particularly pub-drinking occasions where beer was often dominant, such as *Male friends at the pub, Quiet drink at the pub* and *Quiet drink at home alone and with friends in the local*.

**Fig. 3:**
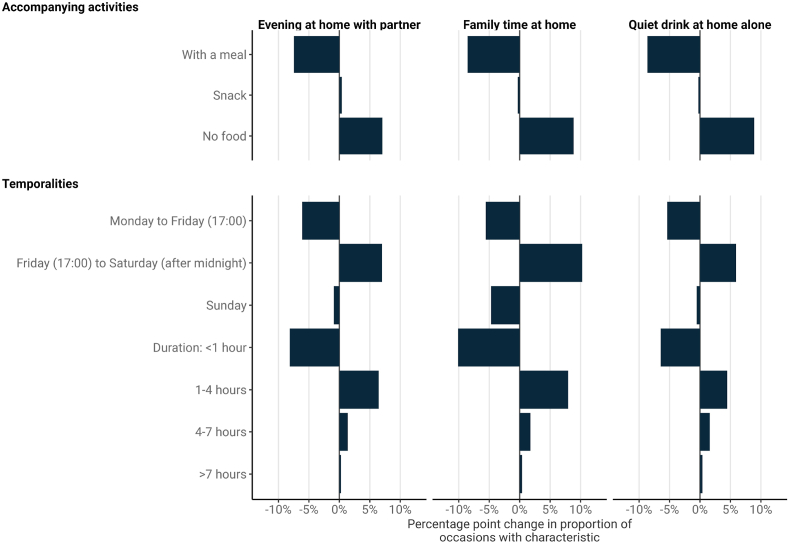
Selected changes in the characteristics of routine home-drinking occasion types, 2009–2019.

In off-trade only occasions, the changes in characteristics for *Quiet drink at home alone, Family time at home* and *Evening at home with partner* occasions suggest performances of these practices are moving away from shorter, weekday occasions where respondents consumed wine with meals and towards longer, weekend occasions where they consumed spirits without a meal (Fig. 3). For example, the proportion of these occasion types that included a meal decreased by between 7.4pp and 8.6pp, the proportion lasting less than an hour decreased by between 6.4pp and 10.1pp, and the proportion taking place on a weekday decreased by between 5.4pp and 6.1pp. In contrast, the proportion taking place on a Friday evening or Saturday increased by between 5.9pp and 10.3pp.

**Fig. 4:**
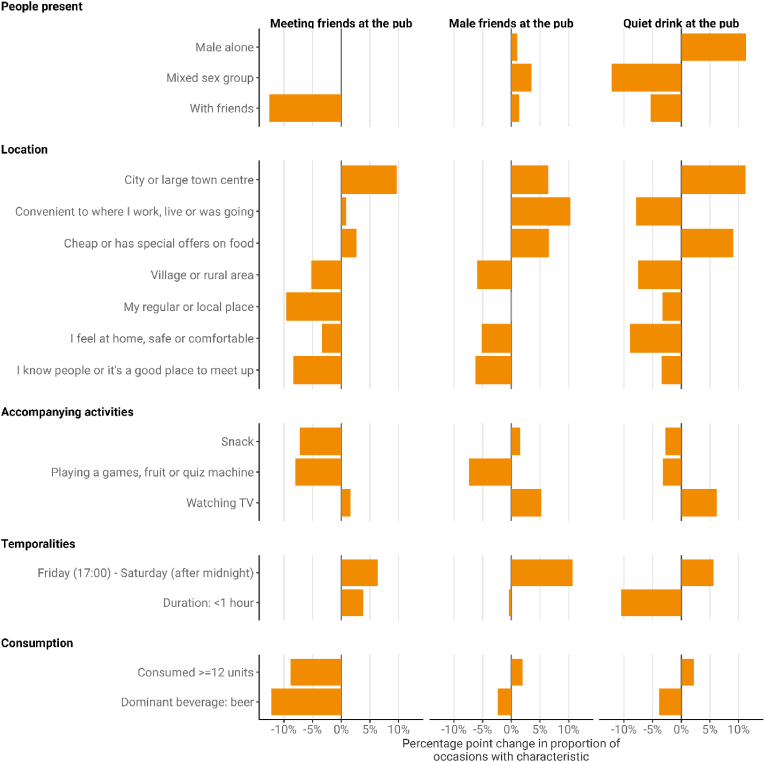
Selected changes in the characteristics of pub-drinking occasion types, 2009–2019.

**Fig. 5:**
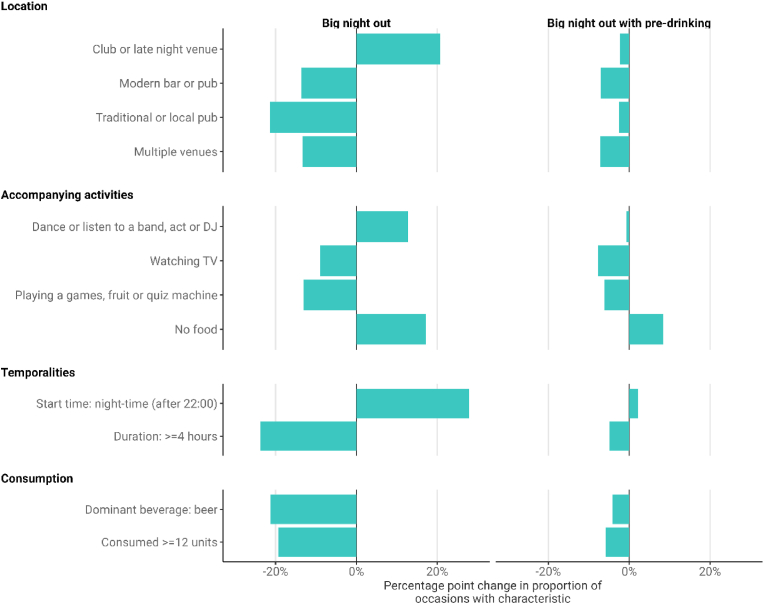
Selected changes in the characteristics of Big Night Out occasion types, 2009–2019.

There were varying degrees of change across the three main pub drinking practices (i.e. *Meeting friends at the pub, Males friends at the pub* and *Quiet drink at the pub;* Fig. 4). The proportion of occasions taking place on a Friday night or Saturday increased across all three types by between 5.6pp and 10.7pp. All of these occasion types also became more urbanised, with the proportion of occasions occurring in rural or village locations decreasing by between 5.3pp and 7.5pp, while the proportion in city centres increased by between 6.4pp and 11.2pp. Participants were also less likely to give reasons for venue choice that suggested familiarity (e.g. a local pub, knowing people there, feeling at home), and instead emphasised the venue's convenience or its cheapness. Other changes were limited to specific occasion types. *Meeting friends at the pub* occasions changed the most, with decreases in the proportion of occasions involving snacks (−7.3pp), games or quiz machines (−8.0pp), beer as the dominant beverage (−12.2pp), consuming 12 or more units (−8.9pp) and friends (−12.5pp), with the latter trend suggesting a more fundamental change in the occasion type. *Quiet drink at the pub occasions* also saw significant changes, with the proportion involving a mixed sex group decreasing by 12.2pp and the proportion involving friends decreasing by 5.4pp, while the proportion involving a male on his own increased by 11.3pp. These occasions also became longer and involved more TV watching. Finally, *Male friends at the pub* occasions changed less, with an increase in TV watching (+5.2pp), decrease in games or quiz machine use (−7.4pp) and consumption of snacks (+10.7pp) but few other specific changes (see Fig. 5).

On-trade only and mixed-trade big night out occasions changed markedly over the study period. For example, the proportion of on-trade only *Big nights out* involving a night club increased by 20.7pp (although figures are subject to volatility), while the proportion involving a traditional pub or modern bar decreased by 21.4pp and 13.6pp respectively. In line with this, these occasions also became more likely to involve dancing or listening to a band, live act or DJ, and less likely to involve barroom games or a snack or meal. The timing of *Big nights out* also changed, with the proportion starting after 22:00 increasing by 27.8pp (subject to volatility) and the proportion lasting more than 4 h decreasing by 23.8pp. They also saw lighter drinking, with the proportion involving consumption of 12 or more units of alcohol decreasing by 19.3pp. As noted above, the proportion where spirits was the dominant beverage also increased by 27.1pp while the proportion with beer as the dominant beverage decreased by 21.3pp. Changes were less marked for the mixed-trade *Big night out with preloading* occasions, although some changes are still visible. For this occasion type we compared between 2009 and the average of results for 2016 and 2017 due to volatility in latent class models for later years. The main changes related to reduced activity in the on-trade before visiting a nightclub. For example, the proportion of *Big night out with preloading* occasions involving a modern bar or multiple venues decreased by 7.0pp and 7.1pp respectively, the proportion involving watching TV decreased by 7.7pp, and the proportion involving no food increased by 8.4pp.

Beyond these changes, the latent class model results show a mixture of more difficult to interpret changes and some evidence of stability in particular occasion types. For example, *Extended occasions (on-trade)* and *Extended occasions (mixed-trade)* show substantial evidence of change over time, although this is not consistent across the two trade sectors. In contrast, other occasion types, such as *Family meal, Meal with friends,* and *Going out with partner* show only modest evidence of change beyond those shifts common to many occasion types, such as a decline in the proportion of occasions taking place in village/rural locations or on weekdays, and where the dominant beverage is wine or beer rather than spirits. However, despite the label we assigned to them, all of these occasion types became less likely to involve a meal, with the proportion doing so decreasing by between 8.8pp and 11.8pp.

## 4 Discussion

The results above suggest that British drinking culture and the practices that comprise it showed aspects of both stability and change between 2009 and 2019. The overall distribution of drinking occasions (i.e. practice performances) across occasion types (i.e. practice formats) remained relatively stable despite evidence of two modest shifts: (i) away from drinking with a partner and towards drinking alone in the off-trade and (ii) away from the stereotypical *Big night out* and towards the more nebulous *Extended occasions* in the on-trade. Changes in the performance of drinking practices were complex and open to multiple interpretations, but we highlight four robust changes. First, spirits were increasingly likely to be the dominant beverage in performances of most practices. The extent to which spirits displaced wine and beer as the dominant beverage varied substantially, but those practices associated with higher levels of alcohol consumption were particularly affected (e.g. *Big nights out* and *Off-trade get togethers*). Second, there was evidence of shifts in home drinking consistent with a move away from routine wine-drinking with meals on weekdays. Third, all pub-drinking practices changed to some degree but to differing extents, with *Male friends at the pub* appearing the most stable. Fourth, changes in the on-trade only *Big night out* and the mixed-trade *Big night out with pre-drinking* were consistent with a move away from the stereotypical pub-crawl before visiting a nightclub and towards going directly to the nightclub later in the evening, sometimes after drinking at home first.

This study used a novel quantitative approach to describe change in the predominant drinking practices observed in Great Britain over an eleven-year period. It drew on a highly detailed event-level dataset collected from a large, nationally-representative sample using consistent methods. The analytical process also involved extensive testing to assess the statistical and face validity of the final models. The key limitations are similar to our previous work with this dataset (Ally et al., 2016). In particular, the Alcovision survey draws its sample from an online market research panel and uses a quota sampling method. Although we developed survey weights to improve representativeness, sampling biases may remain. However, it is unclear whether these biases are substantially worse than those arising from broader problems of representativeness present in other alcohol consumption surveys (Rehm et al., 2021). The Alcovision questionnaire is also designed for market research purposes and therefore does not address key public health concerns, such as negative or intoxication-related motivations for alcohol consumption or harms arising from drinking occasions. Nonetheless, there is no alternative dataset in Britain or, to our knowledge, internationally that would permit analyses of changes in drinking practice at this level of detail. A key limitation of the present analysis is the instability in the model results from 2017 onwards for some latent classes. There are two potential explanations for this. First, sampling biases arising from using multiple panels from 2017 onwards and the loss of July 2017 data, although our weighting procedure should mitigate these problems. Second, small numbers of occasions in some classes relative to the number of variables included in the latent class model, which make parameter estimates sensitive to small variations in the data. This is particularly problematic for the mixed-trade model, where the classes are already more difficult to interpret. Developing an alternative analytical approach to analysing mixed-trade occasions may be useful in future work.

Our findings provide new evidence to explain familiar alcohol consumption trends. Three examples demonstrate this. First, researchers and commentators have increasingly paid attention to habitual drinking in domestic settings (MacLean et al., 2022; Mäkelä et al., 2022), and particularly the popular notion of ‘wine o'clock’ (Wright et al., 2022). Our findings suggest habitual home drinking became less common between 2011 and 2019 and highlight how new materials and connections between those materials and other practice elements may have contributed to this. For example, the emergence of new spirit-based drinks marketed towards women and for relaxed home consumption (e.g. flavoured gins) may have prompted a growing preference for spirits drinking within low-key domestic occasions, as seen in sales data showing the declining market share of wine relative to spirits in the off-trade (British Beer and Pub Association, 2022). This may have disrupted routinised connections between drinking, food and mealtimes because spirits accompany food less well than wine. Such disruptions may then support conscious or unconscious reductions in weekday drinking.

Second, on-trade drinking in Britain changed substantially across recent decades, with the rise and fall of club culture, expansion and diversification of the restaurant sector and the decline of traditional pubs (Angus et al., 2017; Warde, 2016). While some practices changed markedly with these trends, others showed greater resilience. *Male friends at the pub*, *Going out with partner*, and meal-based on-trade practices changed only incrementally and to accommodate major trends (e.g. the growth of spirits drinking), suggesting that these practice formats each have a tightly interconnected set of core elements that limit individuals' scope to adapt their performances of the practice in response to external changes. This is reflected, for example, in Emslie et al.’s (2013) account of the tightknit but negotiable connections between pubs, beer, friendship and hegemonic working class masculinity, in their study of middle-aged men's drinking.

Third, the emergence and characteristics of pre-drinking as an intoxication-oriented drinking practice are well-documented (Labhart et al., 2013). However, the extent of its impact on the ‘Big night out’ requires further investigation. Our findings suggest a substantial disconnect between the traditional *Big night out* as ‘pub crawl then nightclub’ and more recent *Big nights out*. Even when people do not preload in this practice they increasingly omit key elements, such as the dominance of beer, visiting multiple venues and starting early- or mid-evening. This may help to explain the concentration of the night-time economy into a smaller number of late night venues (Angus et al., 2017).

Future research should seek to further unpack and explain the changes described above. This may include qualitative exploration of the links between changes in particular elements and changes in practice formats, focused statistical analyses of individual practices and the sociodemographic characteristics of those participating in them, or comparative research to understand shifting national drinking cultures in new countries (e.g. Mäkelä & Härkönen, 2022). Notably, many of the practice formats we highlight are understudied and may merit greater scrutiny to understand their characteristics and significance. Finally, analysing how practice-level changes link to trends in alcohol-related harm and inequalities in those harms can help to develop understandings of the aetiology of alcohol problems that go beyond standard epidemiological accounts and speak directly to social and commercial determinants of health via analysis of products, marketing, geographies and normative cultures (Meier et al., 2017).

## 5 Conclusion

British drinking culture between 2009 and 2019 was characterised by a mixture of stability and change. Although the practices comprising the culture were largely stable, changing performances of practices included shifts away from habitual home drinking of wine, substantial transformation of the ‘Big night out’, and persistence in some but not all aspects of pub drinking and meal-based drinking practices.

## Primary funding

This work was supported by 10.13039/501100000269Economic and Social Research Council Grant Number ES/R005257/1. For the purpose of open access, the author has applied a Creative Commons Attribution (CC BY) licence* to any Author Accepted Manuscript version arising.

## Ethical statement

This study received ethical approval from the University of Sheffield ethics committee (017,910).

## Author statement

**John Holmes**: Conceptualisation, Methodology, Data curation, Writing – Original draft, Supervision, Funding acquisition.

**Alessandro Sasso**: Methodology, Formal analysis, Data curation, Writing – Original draft.

**Mónica Hernándes Alava**: Methodology, Formal analysis, Writing - Review & Editing, Funding acquisition.

**Abigail K Stevely**: Data curation, Writing - Review & Editing.

**Alan Warde**: Conceptualisation, Writing - Review & Editing, Funding acquisition.

**Colin Angus**: Writing - Review & Editing, Visualisation.

**Petra S Meier**: Conceptualisation, Methodology, Writing - Review & Editing, Supervision, Project administration, Funding acquisition.

## Declarations of competing interest

We have no conflicts to declare. Use of the Alcovision data is allowed under the terms of the contract and nondisclosure agreement between Kantar and the University of Sheffield. This requires research outputs to be submitted to the data provider ahead of publication. The data provider's right to request changes is limited to matters of accuracy regarding descriptions of the data and data collection methods. The data provider played no further role in the research process, including in conception, design, analysis, interpretation, write-up or the decision to publish. The 10.13039/501100000858University of Sheffield, and later the University of Glasgow, purchased the data under license from Kantar using funds provided at different time points by the Medical Research Council, 10.13039/501100000269Economic and Social Research Council, Public Health Scotland and the 10.13039/501100000858University of Sheffield.

## Data Availability

The authors do not have permission to share data.

## References

[bib1] Ally A., Lovatt M., Meier P., Brennan A., Holmes J. (2016). Developing a social practice-based typology of British drinking culture in 2009-2011: Implications for alcohol policy analysis. Addiction.

[bib2] Angus C., Holmes J., Maheswaran R., Green M.A., Meier P., Brennan A. (2017). Mapping patterns and trends in the spatial availability of alcohol using low-level geographic data: A case study in England 2003–2013. International Journal of Environmental Research and Public Health.

[bib3] Blue S., Shove E., Carmona C., Kelly M.P. (2014).

[bib4] British Beer and Pub Association (2022).

[bib5] Cohn S., Lynch R. (2017). Falling into a routine: From habits to situated practices. Sociology of Health & Illness.

[bib6] Emslie C., Hunt K., Lyons A. (2013). The role of alcohol in forging and maintaining friendships amongst Scottish men in mid-life. Health Psychology.

[bib7] Hargreaves T. (2011). Practice-ing behaviour change: Applying social practice theory to pro-environmental behaviour change. Journal of Consumer Culture.

[bib8] Hennell K., Piacentini M., Limmer M. (2020). Exploring health behaviours: Understanding drinking practice using the lens of practice theory. Sociology of Health & Illness.

[bib9] Hennell K., Piacentini M., Limmer M. (2021). ‘Go hard or go home’: A social practice theory approach to young people’s ‘risky’alcohol consumption practices. Critical Public Health.

[bib10] Holmes, J., Sasso, A., Hernández Alava, M., Borges Neves, R., Stevely, A., Warde, A., & Meier, P. (Forthcoming). The distribution of alcohol consumption and heavy episodic drinking across British drinking occasions in 2019: an event-level latent class analysis of drinking diary data. 10.1016/j.drugpo.2024.104414 38588637

[bib11] Keane H., Weier M., Fraser D., Gartner C. (2017). ‘Anytime, anywhere’: Vaping as social practice. Critical Public Health.

[bib12] Labhart F., Graham K., Wells S., Kuntsche E. (2013). Drinking before going to licensed premises: An event-level analysis of predrinking, alcohol consumption and adverse outcomes. Alcoholism: Clinical and Experimental Research.

[bib13] MacLean S., Room R., Cook M., Mugavin J., Callinan S. (2022). Affordances of home drinking in accounts from light and heavy drinkers. Social Science & Medicine.

[bib14] MacLean S., Savic M., Pennay A., Dwyer R., Stanesby O., Wilkinson C. (2019). Middle-aged same-sex attracted women and the social practice of drinking. Critical Public Health.

[bib15] Mäkelä P., Härkönen J. (2022). When tides turn: How does drinking change when per capita alcohol consumption drops?. Addiction Research and Theory.

[bib16] Mäkelä P., Kumpulainen P., Härkönen J., Lintonen T. (2022). Domestication of drinking: A survey study of changes in types of drinking occasions during periods of increasing and decreasing alcohol consumption in the 2000s in Finland. Addiction.

[bib17] Maller C.J. (2015). Understanding health through social practices: Performance and materiality in everyday life. Sociology of Health & Illness.

[bib18] Meier P.S., Warde A., Holmes J. (2017). All drinking is not equal: How a social practice theory lens could enhance public health research on alcohol and other health behaviours. Addiction.

[bib19] Nyemcsok C., Pitt H., Kremer P., Thomas S.L. (2022). Viewing young men’s online wagering through a social practice lens: Implications for gambling harm prevention strategies. Critical Public Health.

[bib20] Oldham M., Holmes J. (2018).

[bib21] Public Health Scotland (2022).

[bib22] Rehm J., Kilian C., Rovira P., Shield K.D., Manthey J. (2021). The elusiveness of representativeness in general population surveys for alcohol. Drug and Alcohol Review.

[bib23] Room R. (1992). The impossible dream? Routes to reducing alcohol problems in a temperances culture. Journal of Substance Abuse.

[bib24] Savic M., Room R., Mugavin J., Pennay A., Livingston M. (2016). Defining “drinking culture”: A critical review of its meaning and connotation in social research on alcohol problems. Drugs: Education, Prevention & Policy.

[bib25] Schatzki T.R., Cetina K.K., Savigny E.V. (2001).

[bib26] Shove E. (2010). Beyond the ABC: Climate change policy and theories of social change. Environment and Planning A: Economy and Space.

[bib27] Shove E., Pantzar M., Watson M. (2012).

[bib28] Southerton D. (2013). Habits, routines and temporalities of consumption: From individual behaviours to the reproduction of everyday practices. Time & Society.

[bib29] Spotswood F., Chatterton T., Tapp A., Williams D. (2015). Analysing cycling as a social practice: An empirical grounding for behaviour change. Transportation Research Part F: Traffic Psychology and Behaviour.

[bib30] Spotswood F., Gurrieri L. (2022).

[bib31] Spotswood F., Nobles J., Armstrong M. (2021). “We’re just stuck in a daily routine”: Implications of the temporal dimensions, demands and dispositions of mothering for leisure time physical activity. Sociology of Health & Illness.

[bib32] Spotswood F., Shankar A., Piwek L. (2020). Changing emotional engagement with running through communal self-tracking: The implications of ‘teleoaffective shaping’ for public health. Sociology of Health & Illness.

[bib33] Stevely A.K., Holmes J., Meier P.S. (2021). Combinations of drinking occasion characteristics associated with units of alcohol consumed among British adults: An event-level decision tree modeling study. Alcoholism: Clinical and Experimental Research.

[bib34] Tein J.-Y., Coxe S., Cham H. (2013). Statistical power to detect the correct number of classes in latent profile analysis. Structural Equation Modeling: A Multidisciplinary Journal.

[bib35] Thurnell-read T. (2018). The embourgeoisement of beer: Changing practices of ‘Real Ale’consumption. Journal of Consumer Culture.

[bib36] Twine R. (2015). Understanding snacking through a practice theory lens. Sociology of Health & Illness.

[bib37] Warde A. (2016).

[bib38] Wright C.J., Miller M., Kuntsche E., Kuntsche S. (2022). ‘What makes up wine o’clock? Understanding social practices involved in alcohol use among women aged 40–65 years in Australia. International Journal of Drug Policy.

