# Supplementary material for: Change and stability in British drinking practices and culture between 2009 and 2019: A longitudinal latent class analysis of drinking occasions
Source: SSM Popul Health. 2023 Nov 4;24:101548. doi: 10.1016/j.ssmph.2023.101548 (PMC10682034; doi:10.1016/j.ssmph.2023.101548)
Supplement: Multimedia component 1 [file mmc1.docx]

**APPENDIX A**

Contents

[1. Weighting procedure 2](#_Toc136084167)

[2. Latent class model fitting results 3](#_Toc136084168)

[3. Summary of drinking occasion typology for Great Britain, 2019 5](#_Toc136084169)

[4. Proportion of occasions within each type by trade sector in 2009 and 2019 6](#_Toc136084170)

# Weighting procedure

The use of quota sampling may lead to selective samples given that the probability of selecting individuals is unknown. The researcher can employ post-stratification weighting to adjust for differences between a targeted population and the observed sample characteristics. Weighting consists of dividing the sample population into post-stratification groups defined by specific control variables (age, sex, region, etc.) and applying a multiplicative factor so that the distribution of control variables for each subgroup resembles that of the target population. In practice, higher weights are computed and assigned to groups of individuals that are under-represented in the sample and smaller weights are assigned to over-represented groups. There are two common methods for computing sample weights.^1^ When the joint probabilities of the target variables are known, *cell-level* weights are calculated for each interlocking cell (e.g. the proportion of women over 65 years old, living in Scotland, and with social grade C1) to achieve the corresponding targets. If the joint probabilities are unknown, an iterative process known as *raking* or *rim weighting* is employed to force the marginal distributions of auxiliary variables (strata) to conform to the joint distribution of the targeted population. Given the non-probabilistic sample design of Kantar Alcovision and the over-representation of targeted categories such as Scotland and 18-24 year old individuals, a raking approach is used in the present analysis.

The general procedure of raking is the following: a weight is applied to each individual in the sample such that the weighted distribution of the first control variable matches the distribution of the same variable in the specified target population. Subsequently, an algorithm readjusts the (weighted) distribution of the second variable to match the target population. This is then repeated for all of the other variables considered. Finally, the adjustment process is reiterated N times until the marginal distribution of all control variables has been perfectly matched with the targets. An advantage of using this raking approach is to reduce bias (i.e. deviation between sample and population means of observed characteristics). However, this may come with a penalty as weight calibration may also lead to an increase in the standard error of sample means.^1^ Nevertheless, the benefits of reducing the bias is generally believed to outweigh the cost of an increase in sampling error.^2^

The present analysis uses raking to match the UK Census population profile on three dimensions: social grade, geographic region, and age-sex groups. To avoid weights with very high values, we follow,^3^ who suggest collapsing categories of the control variables such that each category adds up to at least 5% of the population units. The raking procedure is conducted in Stata (version 15) with the command *ipfraking* implemented by Kolenikov.^1^

# Latent class model fitting results

The information below relates to estimate of latent class models for 2019. Information for models from 2009 to 2018 is available on request.

*Figure A.1: Latent class model fit statistics for off-trade only models with two to eight classes.*

*Figure A.2: Latent class model fit statistics for on-trade only models with two to eight classes.*

*Figure A.3: Latent class model fit statistics for mixed-trade models with two to eight classes.*

| Table A.1 - statistical tests of model restrictions for k vs k-1 classes | | | | | |
| --- | --- | --- | --- | --- | --- |
|  |  |  | **Test type (p-value)** | | |
| **Trade  sector** | **Number of  Classes** | **Number of free  parameters** | **Vuong-Lo-Mendell-Rubin** | **Lo-Mendell-Rubin adjusted LRT** | **Bootstrapped LRT** |
| Off-trade only | 2 | 113 | 0.333 | 0.333 | 0.000 |
| Off-trade only | 3 | 170 | 0.000 | 0.000 | 0.000 |
| Off-trade only | 4 | 227 | 0.000 | 0.000 | 0.000 |
| Off-trade only | 5 | 284 | 0.000 | 0.000 | 0.000 |
| Off-trade only | 6 | 341 | 0.000 | 0.000 | 0.000 |
| Off-trade only | 7 | 398 | 0.000 | 0.000 | 0.000 |
| Off-trade only | 8 | 455 | 0.000 | 0.000 | 0.000 |
| On-trade only | 2 | 173 | 0.000 | 0.000 | 0.000 |
| On-trade only | 3 | 260 | 0.000 | 0.000 | 0.000 |
| On-trade only | 4 | 347 | 0.000 | 0.000 | 0.000 |
| On-trade only | 5 | 434 | 0.000 | 0.000 | 0.000 |
| On-trade only | 6 | 521 | 0.000 | 0.000 | 0.000 |
| On-trade only | 7 | 608 | 0.000 | 0.000 | 0.000 |
| On-trade only | 8 | 695 | 0.000 | 0.000 | 0.000 |
| Mixed-trade | 2 | 207 | 0.000 | 0.000 | 0.000 |
| Mixed-trade | 3 | 311 | 0.000 | 0.000 | 0.000 |
| Mixed-trade | 4 | 415 | 0.000 | 0.000 | 0.000 |
| Mixed-trade | 5 | 519 | 0.000 | 0.000 | 0.000 |
| Mixed-trade | 6 | 623 | 0.533 | 0.534 | 0.000 |
| Mixed-trade | 7 | 727 | 0.768 | 0.768 | 0.000 |
| Mixed-trade | 8 | 831 | 0.819 | 0.819 | 0.000 |

# Summary of drinking occasion typology for Great Britain, 2019

| **Quiet drink at home alone**  19.6% of all occasions  28.5% of off-trade only occasions | **Family time at home**  9.3% of all occasions  13.5% of off-trade only occasions | **Evening at home with partner**  23.5% of all occasions  34.1% of off-trade only occasions |
| --- | --- | --- |
| **Nearly always^1^**: Own home, less than four hours.  **Commonly^2^**: Alone, quiet or regular drink, watching TV, no food, evening, Mon-Fri, Fri-Sat. | **Nearly always:** Mixed sex group, partner or family, own home, less than four hours.  **Commonly:** Quiet or regular drink, watching TV, meal, Fri-Sat. | **Nearly always:** Mixed sex pair, partner, own home, less than four hours.  **Commonly:** Quiet or regular drink, watching TV, meal, evening, Fri-Sat, wine. |
| **Off-trade get together**  16.5% of all occasions  23.9% of off-trade only occasions | **Meeting friends at the pub**  3.8% of all occasions  18.0% of on-trade only occasions | **Male friends at the pub**  2.7% of all occasions  12.9% of on-trade only occasions |
| **Nearly always:** N/A  **Commonly:** Mixed sex group, friends, own or other’s home, sociable, games or leisure activities, meal, less than four hours, evening, Fri-Sat. | **Nearly always:** Mixed sex group.  **Commonly:** Friends, traditional pub, regular or local place, no food, less than four hours, evening, Fri-Sat, beer. | **Nearly always:** Friends, Male group or pair.  **Commonly:** Traditional pub, regular or local place, convenient location, no food, evening, 1-4 hours, Fri-Sat, beer. |
| **Quiet drink at the pub**  2.9% of all occasions  13.8% of on-trade only occasions | **Big night out**  1.3% of all occasions  6.1% of on-trade only occasions | **Extended occasion (on-trade)**  3.0% of all occasions  14.4% of on-trade only occasions |
| **Nearly always:** N/A  **Commonly:** Male alone, traditional pub, convenient place, no food, Mon-Fri, beer. | **Nearly always:** Evening or nighttime.  **Commonly:** Mixed sex group, friends, club, city centre, clubbing or night out, having a laugh, live music, no food, 1-4 hours, Fri-Sat, spirits. | **Nearly always:** N/A  **Commonly:** Mixed sex group, friends, child present, multiple venues, traditional pub, quality of food and drinks, games, meal, 1-4 hours, more than four hours, evening, Mon-Fri, Fri-Sat. |
| **Family meal**  2.3% of all occasions  11.2% of on-trade only occasions | **Meal with friends**  2.3% of all occasions  10.8% of on-trade only occasions | **Going out with partner**  2.6% of all occasions  12.7% of on-trade only occasions |
| **Nearly always:** N/A  **Commonly:** Mixed sex group, family, food pub or restaurant, quality of food or drinks, meal, lunchtime or afternoon, beer. | **Nearly always:** N/A  **Commonly:** Mixed sex group, friends, food pub or restaurant, quality of food or drinks, meal, 1-4 hours, Mon-Fri, Fri-Sat. | **Nearly always:** Mixed sex pair, partner, less than four hours.  **Commonly:** Food pub or restaurant, quality of food or drinks, convenient location, having time for partner, meal, no food, evening, Fri-Sat, beer. |
| **Big night out with pre-drinking**  2.3% of all occasions  22.6% of mixed-trade occasions | **Quiet drink at home and with friends in the local.**  4.1% of all occasions  39.2% of mixed-trade occasions | **Extended occasion (mixed-trade)**  3.9% of all occasions  38.1% of mixed-trade occasions |
| **Nearly always:** Friends, own or other’s home.  **Commonly:** Mixed sex group, city centre, pre-drinking, having a laugh, games or leisure activities, no food, 1-4 hours, more than four hours, evening, Fri-Sat, off🡪on. | **Nearly always:** N/A  **Commonly:** Mixed sex pair, partner, friends, own home, traditional pub, chilling out, watching TV, 1-4 hours, evening, off🡪on, on🡪off, beer. | **Nearly always:** N/A  **Commonly:** Mixed sex group, family, friends, partner, child present, own home, meal, 1-4 hours, more than 4 hours, lunchtime or afternoon, evening or night-time, off🡪on. |
| ^1^Nearly always: Conditional probability ≥ 0.9. ^2^Commonly: Conditional probability is ≥ 0.4 and < 0.9. Conditional probabilities for categories are sometimes combined for interpretability. See Appendix Table A2.2 for full results | | |

# Proportion of occasions within each type by trade sector in 2009 and 2019


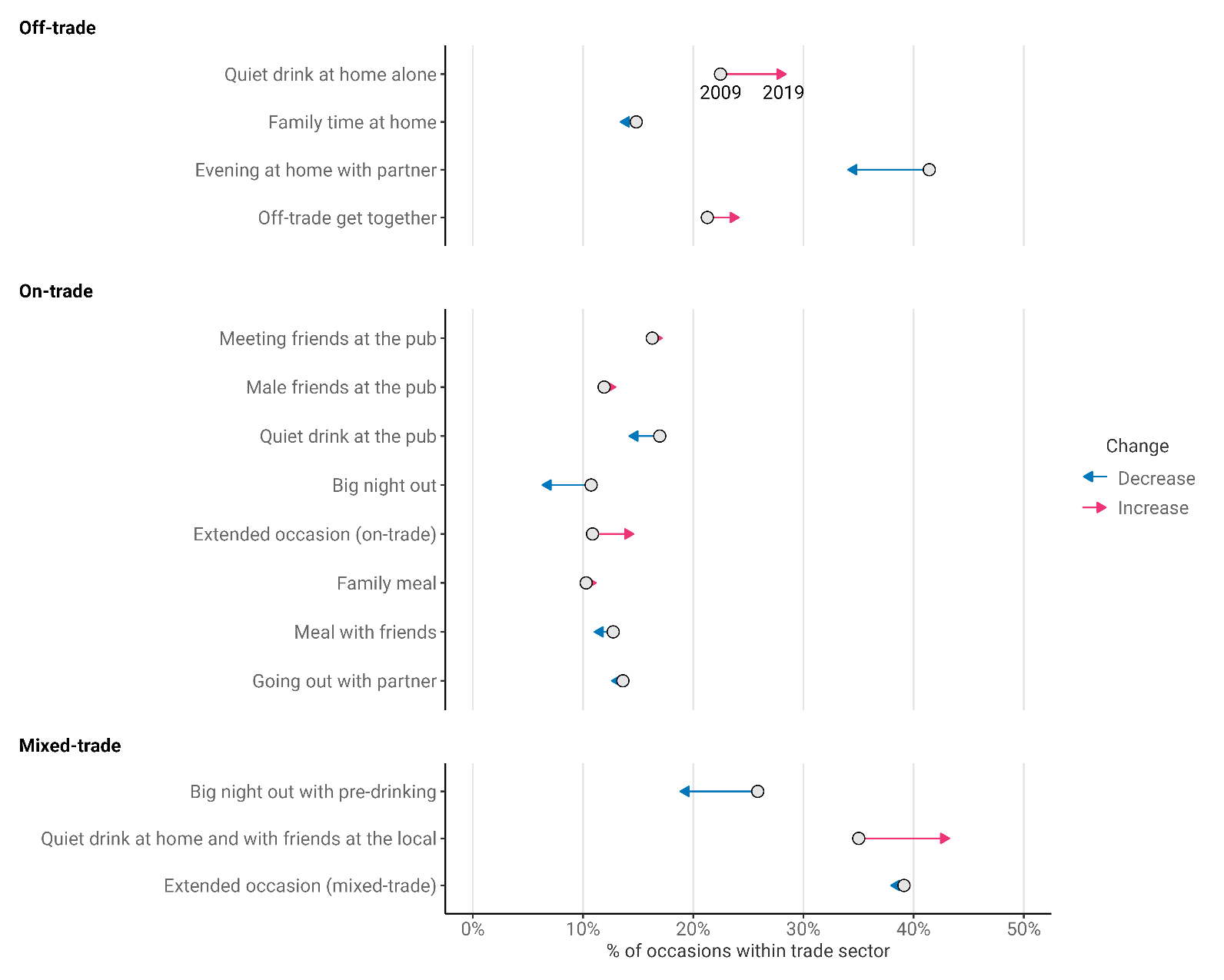


**Appendix references**

1. Kolenikov S. Calibrating survey data using iterative proportional fitting (raking). *The Stata Journal* 2014; **14**(1): 22-59.

2. Baxter M. A better rim weighting algorithm. *International Journal of Market Research* 2016; **58**(4): 621-34.

3. Battaglia MP, Hoaglin DC, Frankel MR. Practical considerations in raking survey data. *Survey Practice* 2009; **2**(5): 2953.
